# Metastatic Head and Neck Non-Melanoma Skin Cancer: A Retrospective Analysis of Clinico-Pathologic Features and Reconstructive Approach

**DOI:** 10.3390/jcm14186650

**Published:** 2025-09-21

**Authors:** Victor Vlad Costan, Otilia Boișteanu, Delia Gabriela Ciobanu Apostol, Ștefan Vasile Toader, Cristina Colac Boțoc, Alin Gabriel Colac, Mihai-Liviu Ciofu, Mihaela Paula Toader

**Affiliations:** 1 Department of Oral and Maxillofacial Surgery, Grigore T. Popa University of Medicine and Pharmacy, 700115 Iasi, Romania; 2 Department of Anesthesiology and Intensive Care, Grigore T. Popa University of Medicine and Pharmacy, 700115 Iasi, Romania; 3 Department of Morpho-Functional Sciences I, Faculty of Medicine, “Grigore T. Popa” University of Medicine and Pharmacy, 700115 Iasi, Romania; delia.ciobanu@umfiasi.ro; 4 Discipline of Physiopathology, Grigore T. Popa University of Medicine and Pharmacy, 700115 Iasi, Romania; 5 Dermatology Clinic, University Clinical Railways Hospital, 700115 Iasi, Romania; 6 Discipline of Oral Medicine, Oral Dermatology (Dermato-Venereology), Grigore T. Popa University of Medicine and Pharmacy, 700115 Iasi, Romania

**Keywords:** NMSC, cSCC, head and neck metastases, reconstructive surgery

## Abstract

**Background/Objectives:** Non-melanoma skin cancer (NMSC) is the most common malignancy globally, with cutaneous squamous cell carcinoma (cSCC) posing a significant risk of regional metastasis, especially in high-risk anatomical areas such as the head and neck. While general risk factors for metastasis are well known, few studies have directly compared the clinical and pathological features of synchronous versus metachronous metastatic behavior. This study aimed to evaluate the clinicopathological characteristics and reconstructive implications associated with these two metastatic patterns in head and neck NMSC. **Methods:** We conducted a retrospective observational study of 46 patients with histologically confirmed metastatic NMSC of the head and neck, treated between January 2022 and May 2024 at a tertiary care center. Patients were stratified into synchronous or metachronous metastasis groups. Clinical data, histopathological features, metastatic sites, and surgical approaches were analyzed. Comparative statistics were applied using chi-square and *t*-tests, with significance set at *p* < 0.05. **Results:** Of the 46 patients, 50% had synchronous and 50% had metachronous metastases. The lower lip was the most common primary tumor site in both groups. Perineural and lymphovascular invasion were more frequent in synchronous metastases. Metachronous cases often required more complex reconstructive procedures, including free flap reconstructions and mandibular resections. Patients with metachronous metastases were significantly older (*p* = 0.024), and approximately one-third developed metastases more than four years after initial treatment. **Conclusions:** Head and neck NMSC, particularly involving the lower lip, may exhibit late-onset metastatic potential. Risk-adapted surveillance extending beyond current guidelines is warranted to improve long-term outcomes in high-risk patients.

## 1. Introduction

Non-melanoma skin cancer (NMSC) represents one of the most frequently diagnosed malignancies worldwide, accounting for approximately 30% of all cancers [1]. While NMSC includes a broad spectrum of skin malignancies such as sarcomas, Merkel cell carcinoma, and cutaneous adnexal tumors, the vast majority are keratinocyte-derived neoplasms, namely basal cell carcinoma (BCC) and squamous cell carcinoma (SCC) [2]. BCC constitutes around 80% of NMSC cases, whereas SCC accounts for approximately 20% [3].

The development of NMSC is multifactorial, with risk factors including cumulative ultraviolet (UV) exposure (natural or artificial), fair skin phenotype, immunosuppression, chronic inflammatory conditions, HPV infection, prior skin cancers, and genetic susceptibility [4]. Epidemiological studies consistently show that advanced age and male sex are independently associated with increased NMSC incidence [5].

Genetic alterations play a pivotal role, particularly in BCC pathogenesis. Dysregulation of the Hedgehog signaling pathway, often through mutations in the PTCH1 or SMO genes, is a hallmark molecular event in BCC development. Additional mutations in tumor suppressor genes such as TP53, frequently associated with UV-induced DNA damage, further contribute to tumorigenesis [6,7]. BCC presents in various clinical forms, including superficial, nodular, morpheaform, and basosquamous subtypes. Histologically, BCC subtypes are stratified into low- and high-risk categories based on recurrence potential, with infiltrative, morpheaform, basosquamous, and micronodular variants associated with increased aggressiveness [8,9]. Although metastatic BCC is rare, when present it typically involves lymph nodes, lungs, or bones [10].

SCC, in contrast, often arises from precursor lesions such as actinic keratoses or Bowen’s disease (in situ SCC). Progression involves a series of genetic and epigenetic events, culminating in invasive and potentially metastatic carcinoma. Clinical presentations of SCC include hyperkeratotic plaques, nodules, or non-healing ulcers, frequently found on chronically sun-exposed skin [11]. Well-differentiated SCCs tend to present as firm, scaly nodules, while poorly differentiated tumors often appear as ulcerated, infiltrative lesions. Keratoacanthomas, though considered a variant of SCC by some, demonstrate rapid growth and are typically located on sun-exposed areas of the skin [12]. Histologically, SCC encompasses various subtypes including spindle cell, acantholytic, and adenosquamous forms, each with distinct biological behavior [13]. Unlike BCC, SCC demonstrates a higher potential for metastasis, particularly in tumors that are poorly differentiated, large, deeply invasive, or located in high-risk anatomical regions such as the lower lip, ear, temple, scalp, and periorbital region. Common metastatic sites include regional lymph nodes, lungs, bone, and mediastinum [14]. Of particular clinical significance is the timing of metastatic spread, synchronous metastases are present at the time of diagnosis or shortly thereafter, while metachronous metastases develop during follow-up after a disease-free interval [15]. Understanding this temporal distinction is essential for accurate staging and guiding long-term surveillance.

From a dermatological perspective, early recognition of suspicious lesions, particularly those occurring on high-risk anatomical sites such as the lips, ears, scalp, temple, and periorbital region, is critical. Dermatologists play a central role in initial diagnosis through clinical and dermoscopic evaluation, performing biopsies, and coordinating multidisciplinary care when aggressive or metastatic disease is suspected. Dermoscopic patterns, although more commonly associated with BCC diagnosis, are increasingly being explored in cSCC to aid in distinguishing subtypes and predicting behavior [16]. Moreover, dermatologists are responsible for long-term follow-up, especially in high-risk patients where late (metachronous) metastases can emerge after initial local control. The integration of histopathological findings with clinical vigilance is vital for effective staging and timely intervention.

Despite the high incidence of NMSC detailed data on its metastatic behavior, particularly distinguishing between synchronous and metachronous spread in the anatomically complex head and neck region, remain scarce [17]. While previous studies have identified general risk factors for metastasis, few have directly compared the clinical and pathological features associated with different metastatic timelines [18].

This study aims to fill that gap by retrospectively analyzing a cohort of patients with metastatic head and neck NMSC, focusing on tumor characteristics, anatomical distribution, histological subtypes, and reconstructive needs in synchronous versus metachronous cases. Notably, we highlight the unexpectedly high metastatic potential of lower lip carcinomas and challenge existing perceptions of their risk profile. By quantifying key histopathological features such as perineural and lymphovascular invasion and evaluating the long-term timeline of metachronous spread, our findings support a more nuanced approach to prognosis, surgical planning, and long-term surveillance strategies in high-risk NMSC patients.

## 2. Materials and Methods

We conducted a retrospective observational study that included 46 cases of metastatic NMSC diagnosed between January 2022 and May 2024 in the Department of Oral and Maxillofacial Surgery at the “Saint Spiridon” Emergency Clinical Hospital, Iași, Romania. All cases included were diagnosed based on histopathological evaluation of either surgically excised lesions or incisional biopsies. Specimens were fixed in 10% neutral buffered formalin, embedded in paraffin, and subsequently stained with hematoxylin and eosin (H&E) for microscopic examination.

Inclusion criteria were as follows: patients with histologically confirmed NMSC located on the cutaneous surface of the head and neck region, the presence of concomitant metastases in the same anatomical region (such as regional lymph nodes or salivary glands), and the availability of at least one histopathological evaluation of both the primary tumor and the metastatic site. Exclusion criteria included cases without histologically confirmed regional metastasis, cases in which the primary tumor site could not be identified, and cases where the primary malignancy was malignant melanoma.

**Figure 1:**
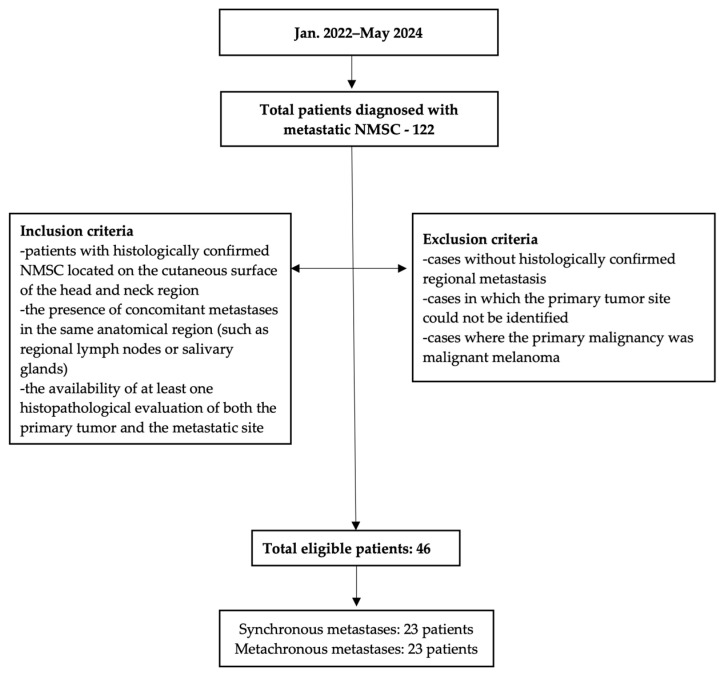
Flowchart of inclusion and exclusion criteria and final cohort distribution.

The patient selection process, including inclusion and exclusion criteria and distribution into synchronous and metachronous metastasis groups, is summarized in a flowchart (Figure 1).

Tumors were classified according to the diagnostic and staging criteria established by the World Health Organization (WHO) and the American Joint Committee on Cancer (AJCC) [19,20]. Histological subtyping was performed to determine the degree of differentiation, presence of perineural or vascular invasion, and margin status, when applicable.

Margin assessment was performed either intraoperatively using frozen sections or postoperatively on permanent paraffin-embedded specimens, depending on the clinical context and surgeon’s preference.

For patients with metachronous metastases, full clinical and pathological data regarding the primary tumor were not always available, as several had been diagnosed or treated at external institutions prior to referral for metastatic disease.

The statistical analysis was carried out in SPSS Inc. (Chicago, IL, USA, version 29.0 for Windows). Data were recorded and analyzed using descriptive and inferential statistical methods. Categorical variables were expressed as frequencies and percentages. To assess potential associations between white lesions and vascular patterns, Pearson’s chi-square test was performed. A *p*-value < 0.05 was considered statistically significant and a *p*-value < 0.01 was considered statistically highly significant.

## 3. Results

**Figure 2:**
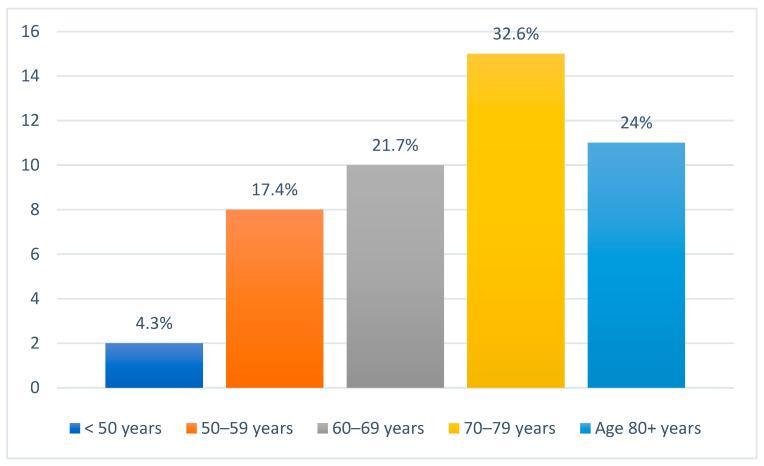
Age distribution of all patients included in the study.

There were 46 patients diagnosed with metastatic NMSC of the head and neck region. Of these, 36 (78.3%) were male and 10 (21.7%) were female. A significant proportion of patients, 31 (67.4%), originated from rural areas, while only 15 (32.6%) were from urban settings. Age distribution revealed that the most affected age group was 70–79 years, comprising 15 patients (32.6%). Patients aged 60–69 years accounted for 10 cases (21.7%), while those aged 80 years or older represented 11 cases (24%). Additionally, 8 patients (17.4%) were between 50 and 59 years old, and only 2 patients (4.3%) were younger than 50 years (Figure 2). In terms of metastatic behavior, 23 patients (50%) presented with synchronous metastases while the other half (50%) developed metachronous metastases (Table 1).

**Figure 3:**
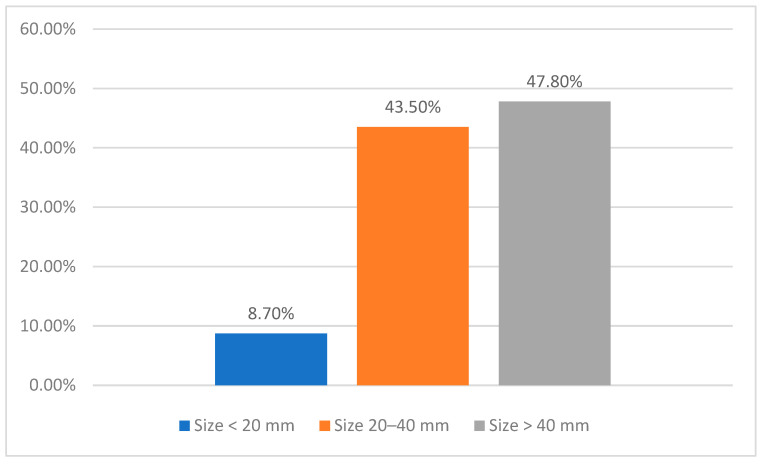
Primary tumor size distribution in the synchronous metastasis cohort.

Among the 23 patients presenting with synchronous metastases, the predominant site of the primary tumor was the lower lip, identified in 78.3% (*n* = 18/24) of cases, followed by a subset of patients (13%, *n* = 3/23) exhibiting extensive tumors affecting multiple anatomical areas and the cheek region in 8.7% (*n* = 2/24). Tumor size was significant, with 47.8% (*n* = 11/24) of lesions measuring greater than 40 mm, 43.5% (*n* = 10/24) between 20 and 40 mm, and 8.7% (*n* = 2/24) were under 20 mm (Figure 3). All cases were diagnosed as cSCC (100%, *n* = 23/23).

**Figure 4:**
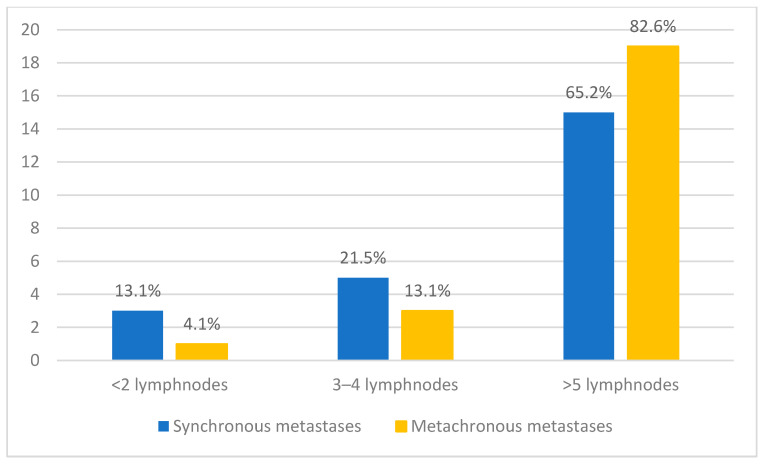
Distribution of patients according to the number of lymph nodes involved, comparing synchronous and metachronous metastases.

Regarding lymph node involvement, most patients in both groups presented with extensive nodal disease. In the synchronous metastasis group, 65.2% (*n* = 15/23) of patients had more than five lymph nodes affected, while 13.1% (*n* = 3/23) had three to four lymph nodes involved, and 13.1% (*n* = 3/23) presented with fewer than two lymph nodes. In the metachronous group, the proportion of patients with more than five lymph nodes involved was even higher, reaching 82.6% (*n* = 19/23) (Figure 4).

Histologically, the distribution of tumor grades showed that G1 (well-differentiated) tumors were the most frequent, accounting for 47.8% of cases (*n* = 11/23), followed by G2 (moderately differentiated) tumors at 43.5% (*n* = 10/23), while G3 (poorly differentiated) carcinomas were the least common, representing 8.7% (*n* = 2/23). Perineural invasion was identified in 52.2% (*n* = 12/23) of patients, and lymphovascular invasion was present in 82.6% (*n* = 19/23). Pathologic staging showed that no tumors were classified as pT1. The most frequent stage was pT2, accounting for 11 cases (47.8%), followed by pT3 with 10 cases (43.5%), while pT4 tumors were identified in 2 cases (8.7%). Regarding nodal classification, N1 metastases were found in 60.9% (*n* = 14/23), N2 in 21.7% (*n* = 5/23), and N3 in 17.4% of cases (*n* = 4/23).

The macroscopic appearance of tumors was primarily ulcerated (95.6%, *n* = 22/23), followed by verrucous (30.4%, *n* = 7/23) and indurated/infiltrative patterns (26%, *n* = 6/23). Less frequent presentations included nodular (8.7%, *n* = 2/23), plaque-like/scaly, crateriform, and pigmented forms (4.3%, *n* = 1/23). Despite the high rate of regional metastases, surgical margins were negative in 100% of cases (Table 1).

The subgroup of 23 patients who developed metachronous metastases presented a distinct clinical profile. The most frequent site of the primary tumor was the lower lip, involved in 60.9% (*n* = 14/23) of cases, followed by the nasal pyramid (13%, *n* = 3/23), upper lip (8.7%, *n* = 2/23), cheek region (8.7%, *n* = 2/23), and ear (4.3%, *n* = 1/23). One patient (4.3%, *n* = 1/23) had an extensive tumor spanning multiple facial regions. All patients in this group were metastasis-free at the time of initial diagnosis, fulfilling the criteria for metachronous spread. The interval between primary tumor treatment and the appearance of metastases varied considerably: 52.2% (*n* = 12/23) of patients developed metastases within 2 years, 17.4% (*n* = 4/23) between 2–4 years, and 30.4% (*n* = 7/23) more than 4 years after the initial surgery. Regarding the location of metastases, lymph nodes were involved in 91.3% (*n* = 21/23) of cases, while salivary gland metastases were observed in 17.4% (*n* = 4/23) bone in 8.7% (*n* = 2/23), and cutaneous metastases in 8.7% (*n* = 2/23). Histopathological grading revealed a predominance of moderately differentiated tumors in 56.5% (*n* = 13/23) of cases, followed by well-differentiated tumors in 34.8% (*n* = 8/23) and poorly differentiated tumors in 8.7% (*n* = 2/23) (Table 1).

Local excision of the primary lesion was performed in 23 cases, accounting for 100% of the cases with available data. Lymph node dissection was performed in a majority of patients, with varying extents: modified radical neck dissection (MRND) was the most common, performed in 14 patients (60.9%), reflecting a balance between oncologic clearance and preservation of vital structures. Selective neck dissection (SND) was used in 5 patients (21.8%). In one of these patients, the lymph node dissection was extended to include the skin overlying the largest lymphadenopathy, located in the lower cheek region (clinical case A, Appendix A). Supraomohyoid dissection, a form of selective dissection involving levels I–III, was performed in 3 patients (13%), and radical neck dissection (RND), the most extensive type, was necessary in only 1 patient (4.3%), suggesting advanced or bulky nodal disease in that case. In terms of reconstruction, local advancement flaps were the preferred method, used in 16 patients (69.6%), indicating that most defects could be managed with adjacent tissue rearrangement. More complex reconstructions were required in a subset of patients: pectoralis major myocutaneous (PMMC) flaps were used in 2 patients (8.7%), sternocleidomastoideus muscle flap associated with a titanium plate to reconstruct the mandible in 1 patient (4.3%) (clinical case B, Appendix A), temporalis muscle flaps in 1 patient (4.3%), and free flap reconstructions were performed in 4 cases (17.4%) (Table 1).

Out of 23 patients, 8.7% (*n* = 2/23) underwent biopsy only. Regarding additional surgical procedures, mandibular resection was performed in 39.1% of cases (*n* = 9/23), while excision of the salivary glands was conducted in 26% (*n* = 6/23). Lymph node dissection was performed in the majority of patients, with MRND being the most common technique, accounting for 69.7% (*n* = 16/23) of cases. SND was performed in 13% (*n* = 3/23), RND in 4.35% (*n* = 1/23), and supraomohyoid dissection also in 4.35% (*n* = 1/23) of patients. Reconstruction methods varied, with 17.4% (*n* = 4/23) receiving a local advancement flap, 21.8% (*n* = 5/23) a PMMC flap, and 52.1% (*n* = 12/23) a free flap. In only 2 cases (8.7%) the reconstruction did not require a reconstructive procedure (Table 1).

When comparing synchronous and metachronous metastases, no statistically significant differences were observed in terms of gender, environment, primary tumor location, histologic subtype, or degree of differentiation (all *p* > 0.05). However, patients with metachronous metastases were significantly older than those with synchronous metastases (*p* = 0.024). The lower lip was the most frequent tumor site in both groups. Importantly, due to incomplete histopathological and staging data for some patients in the metachronous group, stemming from the retrospective nature of the study and treatment performed in different centers, certain comparisons lacked the statistical power to reach significance (Table 1).

## 4. Discussion

This retrospective analysis offers important insights into the clinical and pathological features of head and neck NMSCs with metastatic potential. Metastatic behavior in these cancers remains underexplored, particularly in relation to the timing between synchronous and metachronous metastases. By comparing synchronous and metachronous metastases across 46 cases, we seek to identify specific clinicopathological features associated with a higher risk of regional spread. We also underscore the diagnostic and therapeutic challenges in this anatomically complex region and highlight emerging strategies for surveillance and treatment.

The demographic profile of our cohort aligns with existing literature: the majority of affected individuals were elderly males from rural areas. This observation is consistent with known risk factors for NMSC, such as chronic UV exposure, which is more frequent in individuals with outdoor occupations or lifestyles typical of rural populations [4]. The most frequently affected anatomical site was the lower lip, a location particularly vulnerable to UV radiation due to its exposure and anatomical structure. While lower lip cSCC is often considered to have a lower metastatic risk compared to other head and neck sites, our findings challenge this assumption [21]. Both synchronous and metachronous metastases were predominantly linked to primary tumors on the lower lip, suggesting that this location may merit more intensive surveillance protocols than previously recommended.

Tumor size emerged as a significant determinant of metastatic behavior. In line with prior studies, lesions larger than 40 mm were most frequently associated with synchronous metastases [22,23]. This supports the AJCC staging guidelines that emphasize tumor size as a critical prognostic factor, particularly in the context of head and neck malignancies where anatomical constraints can facilitate deeper invasion and proximity to lymphovascular structures [20].

Histopathological differentiation remains a cornerstone in risk stratification. Although poorly differentiated tumors have long been recognized as more aggressive and more likely to metastasize, our study shows that moderately and well-differentiated tumors also metastasized [24]. This may reflect the overall lower prevalence of G3 tumors in the population but also raises the possibility that other factors, such as perineural invasion or tumor depth, may outweigh differentiation in predicting metastasis in certain contexts.

Perineural invasion and lymphovascular invasion were both frequently observed and strongly correlated with metastatic spread. These findings echo those of Moore et al. and others, who demonstrated that neurotropism is a key predictor of recurrence and nodal involvement [25]. The high prevalence of perineural invasion in synchronous cases supports the hypothesis that this feature may accelerate early regional dissemination, warranting more aggressive local control and early imaging for nodal involvement.

A key contribution of this study is the distinction between synchronous and metachronous metastases. While synchronous spread may reflect delayed diagnosis or inherently aggressive biology, metachronous metastases, observed even after more than four years in some cases, highlight the long-term metastatic potential of NMSC. This challenges the conventional view that most NMSC-related risk dissipates after two to three years post-treatment [26]. Our findings argue for extended surveillance, especially in patients with known risk factors such as perineural invasion, G2-G3 histology, or tumor depth greater than 4 mm. This is especially relevant for lower lip tumors, where early regional spread may be subtle or initially undetectable by clinical examination alone.

Our study demonstrated a predominance of patients with extensive nodal disease, particularly in the metachronous metastasis group, where most individuals presented with five or more affected lymph nodes. This finding highlights an advanced stage of regional spread, which is known to be a critical determinant of prognosis in cSCC of the head and neck. Fang et al. recently proposed a simplified lymph node staging system categorizing patients into three groups based on nodal burden: 1–2 metastatic nodes (N1), 3–4 nodes (N2), and ≥5 nodes or extranodal extension (N3) [27]. Their analysis showed that increasing nodal involvement was significantly associated with decreased locoregional control and overall survival, with the N3 category representing the highest risk group. In line with these findings, the high proportion of patients in our cohort with five or more metastatic nodes suggests a particularly aggressive disease phenotype and underscores the need for intensive management and close postoperative surveillance in this subgroup. These findings emphasize the importance of thorough preoperative imaging and tailored neck dissection strategies, especially for patients with clinically suspicious nodal disease.

Surgery remains the mainstay of treatment for NMSC [28]. In our cohort, surgical excision with negative margins was successfully performed in all synchronous cases. This reinforces the role of histopathologic margin assessment as a critical intraoperative and postoperative parameter. For larger or high-risk tumors, neck dissection was frequently performed, with MRND being the most common approach. This suggests a growing trend toward preemptive regional control in NMSC cases with concerning features.

The management of cervical lymph nodes in head and neck NMSC is pivotal, as underscored by the comprehensive review by Gogna et al. [29]. The presence of nodal metastases significantly diminishes the five-year survival rate, highlighting the necessity for appropriate neck management strategies. Neck dissections, tailored to the extent of disease and anatomical considerations, range from radical to selective approaches. Indications for elective neck dissection include clinically positive nodes and high-risk pathological features such as tumor size exceeding 2 cm, poor differentiation, perineural, and lymphovascular invasion. Surgical precision is paramount to minimize complications, with particular attention to preserving vital structures and preventing chyle leaks. These considerations are integral to optimizing oncologic outcomes and functional preservation in patients undergoing treatment for head and neck NMSC [30].

The selection of reconstructive technique in our series was closely aligned with the extent of oncologic resection and the anatomical complexity of the defect. Among patients with synchronous metastases, local advancement flaps were employed in more than half of cases, reflecting the predominance of smaller, anterior soft-tissue defects, particularly involving the lower lip and cheek region. These cases allowed for primary or local tissue rearrangement with good cosmetic outcomes and minimal functional compromise. In contrast, patients with metachronous metastases frequently presented with more advanced or recurrent disease requiring extensive resections. Notably, more than one third of these patients underwent partial mandibular resection, and approximately half required free flap reconstruction, a significantly higher proportion than in the synchronous group. The increased need for complex flaps, such as radial forearm or anterolateral thigh free flaps, was primarily driven by bone exposure, intraoral communication, or composite tissue loss. PMMC flaps were utilized in one quarter of metachronous cases, particularly when microvascular expertise was unavailable or when patient comorbidities contraindicated free flap transfer.

Compared to recent large cohort studies of head and neck cancer reconstruction, which report free flap usage rates ranging from 10% to 20% in primary and recurrent tumors, our findings reveal a notably higher reconstructive burden in metachronous NMSC cases [31]. This discrepancy may reflect the tendency for these cases to be diagnosed at more advanced stages due to their delayed and often subclinical progression. Functional restoration, especially for speech, mastication, and oral continence, was a key consideration, particularly in lower facial defects. Although detailed functional outcome metrics were not systematically recorded, the surgical strategy emphasized immediate reconstruction where feasible, with flap choice tailored to ensure sufficient bulk, pliability, and mucosal lining. Aesthetic outcome was also prioritized, particularly in lip and cheek reconstructions, to preserve symmetry and avoid contour deformities. Donor site morbidity was minimal, with no reported flap failures or major reconstructive complications documented in the available surgical notes. Overall, the reconstructive approach followed the principles of the reconstructive ladder, escalating from local to regional and free flaps as dictated by defect complexity, and aligned with current NCCN and oncologic reconstructive guidelines.

The role of adjuvant radiotherapy in NMSC, particularly for high-risk cSCC, is well established. Perineural invasion, close or positive margins, and nodal metastases all constitute indications for radiotherapy. Despite its effectiveness, toxicity remains a concern, particularly xerostomia, mucosal irritation, and late complications such as osteoradionecrosis and trismus. Emerging radioprotective strategies, such as salivary gland-sparing techniques and botulinum toxin injections, are promising adjuncts that merit future exploration [32].

In cases where surgery or radiotherapy is contraindicated or ineffective, systemic therapies play an increasingly important role. Immunotherapy, specifically PD-1 inhibitors such as cemiplimab and pembrolizumab, has revolutionized treatment paradigms for advanced NMSC [33]. Our study underscores the need for broader access to these agents, especially given the limited chemotherapy options and their toxicity profile. Targeted therapies, particularly EGFR inhibitors, may further personalize treatment, especially in tumors with known EGFR overexpression [34]. While our study did not include genetic testing, emerging evidence suggests that somatic mutations in TP53, CDKN2A, NOTCH1, and EGFR may promote aggressive behavior in cutaneous SCC. The implementation of next-generation sequencing into pathology workflows could enable earlier identification of tumors with metastatic potential and allow for biologically informed treatment decisions [35].

Although chemotherapy is traditionally reserved for palliative settings, agents like cisplatin, 5-fluorouracil, and taxanes remain relevant. These drugs, often used in combination with immunotherapy or radiotherapy, can provide meaningful symptom control in selected patients [36].

Beyond these clinical and therapeutic considerations, our findings contribute novel insights to the broader understanding of NMSC metastasis, particularly regarding delayed progression and site-specific risk. The present study introduces several important nuances to conventional understanding by offering a comparative analysis of synchronous and metachronous metastases in head and neck NMSC, focusing specifically on real-world clinical and pathological features. One of the most striking findings is that nearly half of the patients in our cohort developed metachronous metastases, with one third of those occurring more than four years after initial treatment. These results strongly suggest that a subset of patients, particularly those with tumors located on the lower lip or with certain histologic features, remain at risk for metastasis long after the standard surveillance period has concluded. This observation supports a growing body of evidence calling for individualized, risk-adaptive follow-up strategies that extend beyond the traditional two- to three-year timeframe.

Additionally, our study challenges prevailing assumptions about the metastatic risk associated with tumor location. Although the lower lip is not typically classified among the highest-risk sites for regional or distant metastasis, it was the most frequently involved site in both synchronous and metachronous groups in our cohort. This finding implies that lower lip cSCC, especially in older male patients and in the context of sun-exposed, chronically inflamed skin, may warrant reclassification into a higher-risk category in future staging and follow-up guidelines. Notably, the anatomical simplicity of the lip may belie its potential for early subclinical invasion, particularly perineural spread, which is often underrecognized in early stages.

Another important contribution of this study lies in the observation that histological grade alone was not a reliable predictor of metastatic potential. In the metachronous metastasis group, the majority of tumors were well- or moderately differentiated, and yet these lesions eventually gave rise to clinically significant regional or distant disease. This undermines the conventional assumption that poor differentiation is the dominant histological driver of aggressive behavior. Our findings align with recent literature suggesting that features such as perineural and lymphovascular invasion, tumor depth, and molecular alterations may serve as more sensitive predictors of metastatic behavior than differentiation grade alone [37]. This reinforces the need for comprehensive histopathologic assessment beyond the binary distinction between well- and poorly differentiated tumors.

From a therapeutic and surgical standpoint, the burden of disease in metachronous cases also appears to be greater. A significant proportion of these patients required extensive surgical intervention at the time of recurrence, 39.1% underwent partial mandibular resection and 47.8% required microvascular free flap reconstruction, substantially higher than in the synchronous cohort. This finding has two important implications. First, it suggests that metachronous metastases are often diagnosed at a more advanced stage, potentially due to their slow or subtle progression and the cessation of routine follow-up. Second, it highlights the considerable reconstructive and functional demands these cases impose on surgical teams, which must be anticipated during long-term management planning.

In most cases, more complex reconstruction techniques were required for metastatic lymphadenopathy. The decision to perform lymph node dissection followed by reconstruction was made more quickly in the case of synchronous lymphadenopathy. In contrast, for metachronous lymphadenopathy, diagnosis was often delayed, and in these cases, extensive surgical excisions were necessary, significantly reducing patients’ quality of life. Regardless of the complexity of the reconstructive technique may be, restoring aesthetic appearance is difficult, and functional reconstruction often yields suboptimal results. Consequently, the follow-up of patients with facial squamous cell carcinomas must be rigorous and should not necessarily depend on the size of the primary tumor. Often, lesions with subtle postoperative scarring are associated with larger lymphadenopathies that invade the skin, the viscerocranium bones, or even the neurocranium (See Appendix A, Clinical Cases 1 and 2).

The risk-adapted surveillance algorithm proposed in this study significantly expands upon existing recommendations found in major international guidelines, such as those issued by the National Comprehensive Cancer Network (NCCN) and the British Association of Dermatologists (BAD) [38,39]. While these guidelines typically recommend a 2–3 years follow-up for high-risk cSCC, our findings challenge the assumption that the metastatic risk largely resolves within this window. Notably, in our cohort, nearly one-third of metachronous metastases occurred more than four years after primary tumor excision. This late-onset metastatic behavior, particularly in patients with lower lip tumors, supports the extension of surveillance to at least five years in selected high-risk individuals, incorporating annual imaging beyond the third year. To our knowledge, this is one of the few studies providing data-driven evidence to justify prolonged monitoring in head and neck NMSC. A structured overview of this approach is provided in Table 2.

In addition, our algorithm introduces several novel elements not widely represented in the current literature. First, it emphasizes the lower lip as a high-risk anatomical site for both synchronous and delayed metastasis, in contrast to existing models that prioritize locations such as the ear, scalp, and temple. Second, it elevates moderately differentiated tumors into a higher-risk category, based on our observation that such tumors frequently gave rise to regional or distant disease despite not being poorly differentiated. Third, the algorithm incorporates a composite risk model, stratifying patients based on the cumulative presence of adverse features including tumor size >40 mm, perineural or lymphovascular invasion, and histologic grade. This multi-factorial stratification reflects the real-world complexity of risk in head and neck cancer and offers a more nuanced approach than binary classification schemes. Finally, the surveillance model uniquely integrates surgical and reconstructive parameters, acknowledging the substantial burden of delayed metastases on functional and aesthetic outcomes. Collectively, these distinctions support the development of tailored follow-up strategies that better align with the clinical behavior of advanced head and neck NMSC. Our proposed surveillance recommendations are outlined in Table 3. The proposed algorithm is intended as a framework, but clinical judgment remains essential to tailor follow-up to each patient’s condition.

In summary, this study adds meaningful, practice-changing insights to the current literature by providing the first detailed comparison between synchronous and metachronous metastases in head and neck NMSC. It identifies a subset of patients, particularly those with lower lip primaries, perineural invasion, or moderate differentiation, who may benefit from prolonged, intensified surveillance beyond current standards. It also suggests that the traditional reliance on tumor differentiation and short-term follow-up may be insufficient for predicting long-term outcomes. These findings support a more personalized, biologically informed approach to patient monitoring and treatment planning, which is increasingly advocated in contemporary oncologic dermatology.

This study has several limitations. Its retrospective design introduces inherent biases, including selection and referral bias. Data completeness, particularly for patients referred from external institutions, varied. Moreover, the relatively small sample size limits the statistical power of subgroup comparisons. Nonetheless, the study’s strengths lie in its detailed clinicopathological correlations, real-world relevance, and focus on a specific anatomical region with unique functional and aesthetic considerations. Future multicenter prospective studies integrating radiologic surveillance, molecular biomarker assessment, and patient-reported outcomes would offer a more comprehensive and personalized roadmap for managing high-risk head and neck NMSC.

## 5. Conclusions

This study provides a comprehensive comparative analysis of synchronous and metachronous metastases in head and neck non-melanoma skin cancer, highlighting distinct clinical and pathological patterns. Our findings suggest that lower lip cutaneous squamous cell carcinoma may carry a higher metastatic risk than previously recognized, particularly in the presence of perineural or lymphovascular invasion and moderate differentiation. Metachronous metastases often occur years after initial treatment and are associated with a higher reconstructive burden, emphasizing the need for long-term surveillance. These results support a more nuanced, risk-adapted approach to patient follow-up and surgical planning. Incorporating extended monitoring protocols and individualized risk assessment could improve early detection of delayed metastases and optimize both functional and aesthetic outcomes. Future prospective studies are warranted to validate these findings and further refine surveillance algorithms for high-risk NMSC patients.

## Figures and Tables

**Table 1:** Comparative Analysis of Patients with Synchronous vs. Metachronous Head and Neck NMSC Metastases.

Variable	Metachronous (*n* = 23)	Synchronous (*n* = 23)	Total (*n* = 46)	Statistical Test	*p*-Value
**Gender**				Chi^2^ = 0.511	0.475
Male	17 (73.9%)	19 (82.6%)	36 (78.3%)		
Female	6 (26.1%)	4 (17.4%)	10 (21.7%)		
**Residence**				Chi^2^ = 0.890	0.345
Urban	6 (26.1%)	9 (39.1%)	15 (32.6%)		
Rural	17 (73.9%)	14 (60.9%)	31 (67.4%)		
**Age (years)**				t = 2.342	0.024 *
Mean ± SD	74.3 ± 11.54	66.5 ± 10.99	70.4 ± 11.82		
Median (IQR)	75 (66–83)	65 (58–76)	72.5 (60.75–79.25)		
**Primary Tumor Location**				Chi^2^ = 8.500	0.386
Lower lip	14 (60.9%)	18 (78.3%)	32 (69.6%)		
Upper lip	2 (8.7%)	0	2 (4.35%)		
Cheek region	2 (8.7%)	2 (8.7%)	4 (8.7%)		
Nasal pyramid	3 (13%)	0	3 (6.52%)		
Ear	1 (4.35%)	0	1 (2.17%)		
Extensive tumors	1 (4.35%)	3 (13%)	4 (8.7%)		
**Macroscopic appearance**					
Ulcerated	N/A	22 (95.6%)			
Nodular	N/A	2 (8.7%)			
Plaque-like/scaly	N/A	1 (4.3%)			
Indurated/infiltrative	N/A	6 (26%)			
Crateriform/keratoacantoma-like	N/A	1 (4.3%)			
Verrucous	N/A	7 (30.4%)			
Pigmented	N/A	1 (4.3%)			
**Histological Subtype**				Chi^2^ = 1.022	1.000
Cutaneous SCC	22 (95.7%)	23 (100%)	45 (97.8%)		
Basosquamous carcinoma	1 (4.3%)	0	1 (2.2%)		
**pT category**					
pT1	N/A	0			
pT2	N/A	11 (47.8%)			
pT3	N/A	10 (43.5%)			
pT4	N/A	2 (8.7%)			
**N category**					
N1	N/A	14 (60.9%)			
N2	N/A	5 (21.7%)			
N3	N/A	4 (17.4%)			
**Perineural invasion**	N/A	12 (52.2%)			
**Lymphovascular invasion**	N/A	19 (82.6%)			
**Differentiation Grade (G)**				Chi^2^ = 1.259	0.533
G1	7 (31.8%)	11 (47.8%)	18 (40.0%)		
G2	13 (59.1%)	10 (43.5%)	23 (51.1%)		
G3	2 (8.7%)	2 (8.7%)	4 (8.9%)		
**Period of time between initial** **surgery and metastasis**					
<2 years	12 (52.2%)	-			
2–4 years	4 (17.4%)	-			
>4 years	7 (30.4%)	-			
**Metastatic Sites**				Chi^2^ = 2.091	0.489
Lymph nodes	21 (91.3%)	23 (100%)	44 (95.7%)		
Bone	2 (8.7%)	0	2 (4.3%)		
Salivary gland	4 (17.4%)	1 (4.3%)	5 (10.9%)	Chi^2^ = 2.020	0.346
Muscle	1 (4.3%)	0	1 (2.2%)	Chi^2^ = 1.022	1.000
Skin	2 (8.7%)	0	2 (4.3%)	Chi^2^ = 2.091	0.489
**Surgery Type**				Chi^2^ = 2.091	0.489
Biopsy only	2 (8.7%)	0	2 (4.3%)		
Tumor excision	21 (91.3%)	23 (100%)	44 (95.7%)		
**Neck Dissection Type**				Chi^2^ = 1.546	0.672
SND	3 (14.3%)	5 (21.7%)	8 (18.2%)		
MRND	16 (76.2%)	14 (60.9%)	30 (68.2%)		
RND	1 (4.8%)	1 (4.3%)	2 (4.5%)		
Supraomohyoid	1 (4.8%)	3 (13.0%)	4 (9.1%)		
**Reconstruction Method**				Chi^2^ = 13.423	0.004 **
Local advancement flap	4 (19.0%)	16 (69.6%)	20 (45.5%)		
PMMC flap	5 (23.8%)	2 (8.7%)	7 (15.9%)		
Temporalis flap	0	1 (4.3%)	1 (2.3%)		
Free flap	12 (57.1%)	4 (17.4%)	16 (36.4%)		

N/A = not available; SND = selective neck dissection; MRND = modified radical neck dissection; RND = radical neck dissection; PMMC = pectoralis major myocutaneous. * statistically significant, ** statistically highly significant.

**Table 2:** Risk Stratification Model for Surveillance in Head and Neck NMSC.

Risk Level	Risk Factors	Description
**Low risk**	- Tumor size < 20 mm - Location: nonlip, nonear - Histologic grade G1 - No perineural or lymphovascular invasion	Tumors with minimal invasion, small size, low-grade histology, favorable location
**Intermediate risk**	- Tumor size 20–40 mm - High risk anatomical area - Histologic grade G2 - Focal perineural invasion	Tumors with moderate aggressiveness due to size, grade, or anatomical location
**High risk**	- Tumor size > 40 mm - High risk anatomical area - Histologic grade G3 - Extensive perineural or lymphovascular invasion - Bone or salivary gland involvement - History of head and neck malignancy	Tumors with aggressive histology, large size, multiple invasive features, or unfavorable oncologic history

Note: Although risk stratification is based on the cumulative number of adverse factors, the presence of a single major feature (e.g., tumor size > 40 mm, histologic grade G3, or lower lip location) should already be considered at least intermediate risk and warrant closer surveillance.

**Table 3:** Proposed risk-adapted surveillance algorithm for patients with head and neck NMSC.

		Surveillance Duration	Follow-Up	Imaging
**Risk factors present**	Low risk	2–3 years	Every 6–12 months	Annually (years 3–5)
	Intermediate risk	5 years	Every 6 months (years 1–3), annually thereafter	Every 6–12 months (ultrasound or CT)
	High risk	>5 years	Every 3–4 months (first 2 years), every 6 months thereafter	Every 6–12 months for 5 years (cross- sectional imaging CT or MRI)

CT—computed tomography; MRI—magnetic resonance imaging. Follow-up intensity is stratified based on cumulative clinicopathologic risk factors, including tumor location, size, histologic grade, and presence of perineural or lymphovascular invasion. High-risk patients warrant surveillance beyond three years, including periodic imaging.

**Figure A1:**
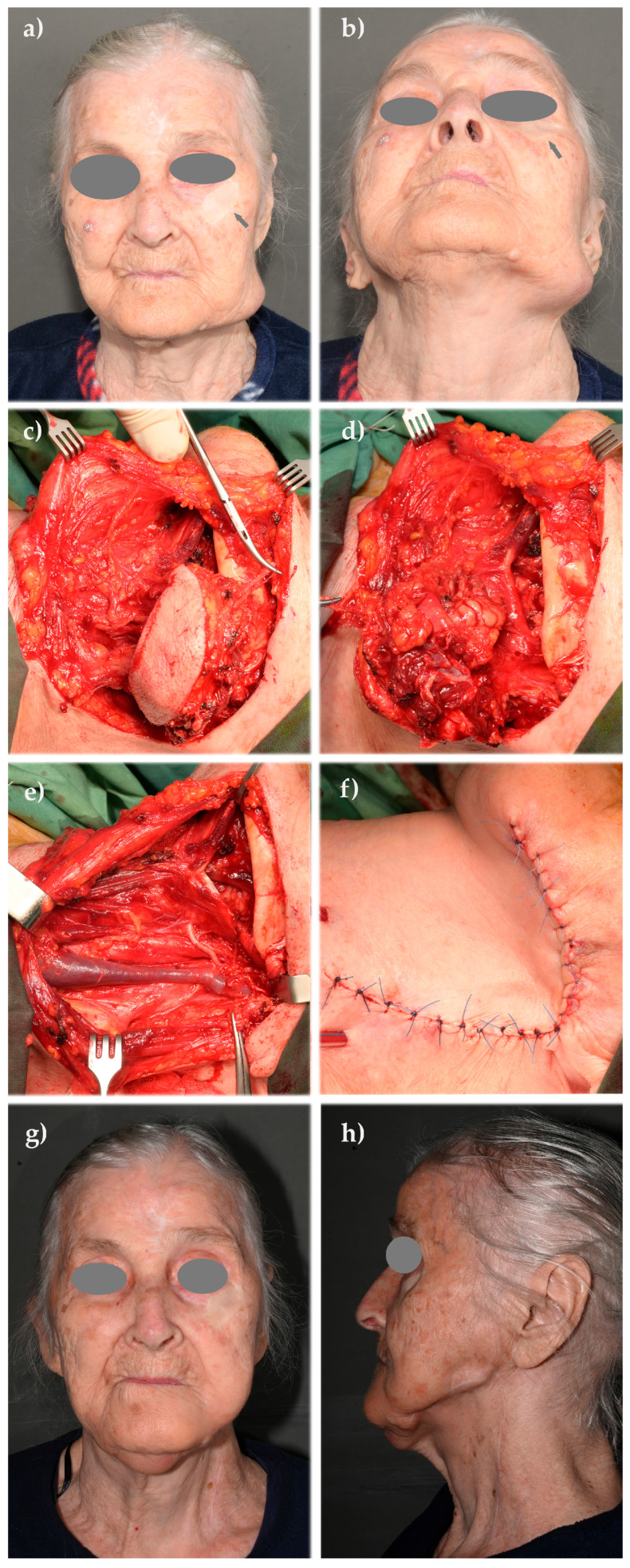
**Clinical case A:** (**a**,**b**) metastatic lymphadenopathy in the lower left buccal and laterocervical region, following a squamous cell carcinoma (SCC) of the cheek operated approximately two years prior to presentation in our department; (**c**) a modified radical neck dissection was necessary, including excision of the skin overlying the tumor; (**d**) clearance of the contents of the left submandibular compartment following skin excision; (**e**) postoperative view of the left laterocervical region showing sacrifice of the spinal accessory nerve and preservation of the sternocleidomastoid muscle (SCM) and internal jugular vein (IJV); (**f**) Reconstructed cutaneous defect using a cervically rotated platysma musculocutaneous flap; (**g**,**h**) one-year postoperative result showing acceptable facial symmetry and flap integration (anterior and lateral views).

**Figure A2:**
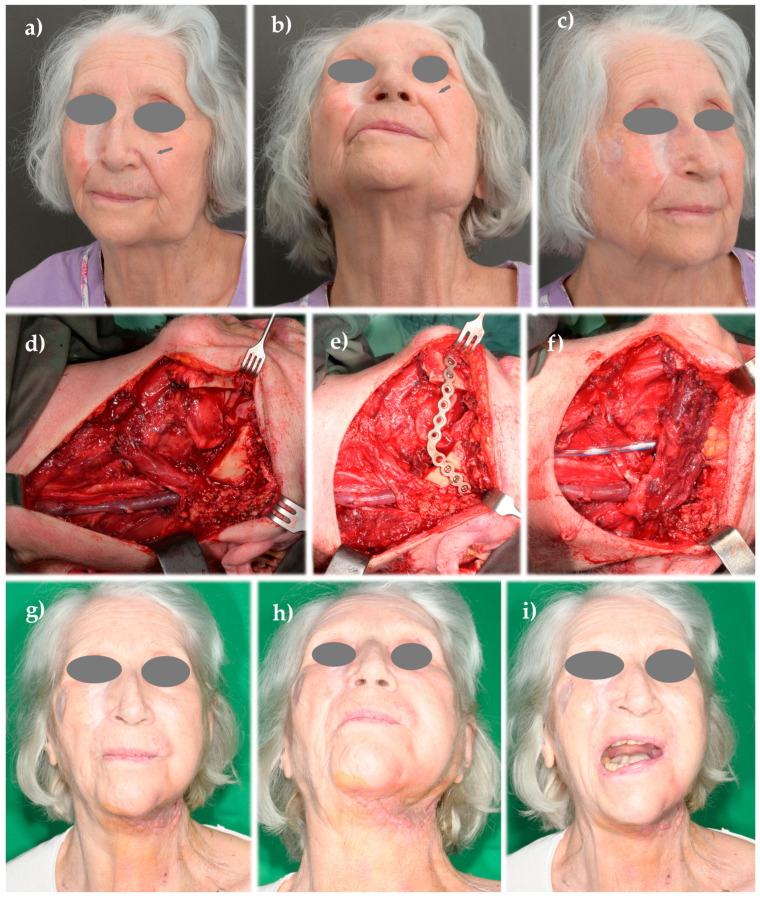
**Clinical case B:** (**a**–**c**) patient with multiple previously excised and locally healed facial cutaneous squamous cell carcinomas (SCCs); the arrow highlights a buccal SCC that, despite leaving a subtle postoperative scar, was followed approximately one year later by a large metastatic lymphadenopathy in the lower cheek, invading the left mandibular body; (**d**) postoperative view following an extended modified radical neck dissection, including segmental mandibular resection; (**e**) mandibular continuity was restored using a titanium reconstruction plate; (**f**) to avoid intraoral or extraoral exposure of the reconstruction plate, it was covered with a muscular flap harvested from the SCM; (**g**–**i**) postoperative appearance showing good facial symmetry.

## Data Availability

All relevant data supporting the findings of this study are contained within the article. As this was a retrospective analysis based on anonymized patient records, no additional datasets are publicly available. Further inquiries can be directed to the corresponding author.

## Abbreviations

AJCC: American Joint Committee on Cancer; BAD: British Association of Dermatologists; BCC: Basal Cell Carcinoma; CT: Computed Tomography; cSCC: Cutaneous Squamous Cell Carcinoma; G1/G2/G3: Tumor Grade 1 (well-differentiated), Grade 2 (moderately differentiated), Grade 3 (poorly differentiated); H&E: Hematoxylin and Eosin; IJV: Internal Jugular Vein; MRI: Magnetic Resonance Imaging; MRND: Modified Radical Neck Dissection; NCCN: National Comprehensive Cancer Network; NMSC: Non-Melanoma Skin Cancer; PNI: Perineural Invasion; PMMC: Pectoralis Major Myocutaneous; RND: Radical Neck Dissection; SCM: Sternocleidomastoid Muscle; SD: Standard Deviation; SND: Selective Neck Dissection; UV: Ultraviolet; WHO: World Health Organization.
